# Maternal cardiovascular adaptation to twin pregnancy: a population-based prospective cohort study

**DOI:** 10.1186/s12884-020-02994-w

**Published:** 2020-05-29

**Authors:** Maria C. Adank, Zoe A. Broere-Brown, Romy Gonçalves, M. Kamran Ikram, Vincent W. V. Jaddoe, Eric A. P. Steegers, Sarah Schalekamp-Timmermans

**Affiliations:** 1 Department of Obstetrics and Gynaecology, Erasmus MC, University Medical Centre Rotterdam, Rotterdam, the Netherlands; 2 Generation R Study Group, Erasmus MC, University Medical Centre Rotterdam, Rotterdam, the Netherlands; 3 Department of Epidemiology, Erasmus MC, University Medical Centre Rotterdam, Rotterdam, the Netherlands; 4 Department of Neurology, Erasmus MC, University Medical Centre Rotterdam, Rotterdam, the Netherlands; 5 Department of Paediatrics, Erasmus MC, University Medical Centre Rotterdam, Rotterdam, the Netherlands

**Keywords:** Blood pressure, Cardiovascular diseases, Pre-eclampsia, Pregnancy, twin, Cardiovascular adaptation

## Abstract

**Background:**

In women with singleton pregnancies, maternal adaptation is considered a stress test for later life cardiovascular disease. The aim of this study was to assess maternal adaptation in women with twin pregnancies compared to women carrying singletons during and after pregnancy.

**Methods:**

This was a population based prospective cohort study of 91 women with twin pregnancies and 8107 women carrying singletons. The association of twin pregnancy and maternal adaptation was examined using regression analyses. In pregnancy, we measured soluble fms-like tyrosine kinase-1 (sFLT-1), placental growth (PGF) factor, systolic (SBP) and diastolic blood pressure (DBP), and the occurrence of pre-eclampsia (PE). After pregnancy, measurements were obtained on SBP and DBP, cardiac function, retinal calibres, intima media thickness and distensibility of the common carotid artery.

**Results:**

sFLT-1 and PGF concentrations were higher in early (13.4 weeks) and mid-pregnancy (20.4 weeks) in women with twin pregnancies compared to women with singleton pregnancies. Women with twin pregnancies had a different DBP pattern in pregnancy. Women with twin pregnancies were more likely to have PE (odds ratio 3.63; 95% CI [1.76 to 7.48]). Six and ten years after pregnancy, no differences in maternal adaptation were observed.

**Conclusions:**

Women with twin pregnancies show an altered adaptation during pregnancy compared to women with singleton pregnancies. This is associated with a substantially increased incidence of PE, but does not lead to persistent altered maternal adaptation years after pregnancy.

## Background

In pregnancy, extensive maternal cardiovascular adaptations lead to proper implantation and placental and fetal growth and development. Women failing to meet the hemodynamic demands of pregnancy are more likely to develop complications such as pre-eclampsia (PE). These women also have an increased risk to develop cardiovascular disease (CVD) later in life [1]. The associations may be explained by persisting endothelial damage of pregnancy and/or a pre-existing unfavourable cardiovascular risk profile [2, 3]. Therefore, pregnancy can be considered as a hemodynamic stress test for long term CVD [3].

Twin pregnancy has been marked as a risk factor of PE [4–6], and may be explained by the higher demand of both foetuses from the mother. This leads to different maternal adaptation to pregnancy [7–9], in response to altered placentation with increased anti-angiogenic factors as soluble fms-like tyrosine kinase-1 (sFLT-1) and pro-angiogenic factors as placental growth factor (PGF). The adaptation to pregnancy is more often inadequate in women with twin pregnancies conceived after assisted reproductive techniques (ART), leading to an even more increased risk of PE [10]. This relation may be explained by the fact that women who conceived after ART are more often older [11–13]. They are also more often nulliparous, which is associated with PE [14].

Given this knowledge on the association of twin pregnancies with PE and the association of PE with CVD, we hypothesize that women with twin pregnancies have an increased risk of future CVD, which is mediated by the increased risk of PE. Therefore, the aim of this study was to investigate maternal adaptation throughout pregnancy and years after pregnancy in women with twin pregnancies compared to women with singleton pregnancies.

## Methods

### Study design

This study was embedded within The Generation R Study. Generation R is an ongoing population-based prospective cohort study from early pregnancy onwards [15]. Mothers with a delivery date between April 2002 and January 2006 were eligible for participation. Standardized examinations were performed within mothers during pregnancy, and within both mother and child 6 and 10 years later. The Medical Ethical Committee of The Erasmus Medical Centre in Rotterdam approved the study (MEC 198.782/2001/31). Written informed consent was obtained from all mothers. Women with a live born singleton or twin pregnancy with at least one measurement available on cardiovascular parameters during and after pregnancy were included in this study. Women who participated more than one time in the cohort were excluded (i.e. with different pregnancies). The total population for analyses comprised 8198 women (Additional file 1: Figure S1). All women were invited to standardized visits three times during pregnancy and 6 and 10 years after pregnancy at the research centre.

### Pregnancy

Early in pregnancy (median 13.4 weeks of gestational age, 90% range 10.5 to 17.2) and in mid-pregnancy (median 20.4 weeks of gestational age, 90% range 18.8 to 23.1), we obtained maternal non-fasting venous blood samples [16, 17]. sFLT-1 and PGF concentrations were determined. sFLT-1 and PGF vary with gestational age and concentrations are therefore not normally distributed. We constructed sFLT-1 and PGF gestational-age-adjusted standardized Multiple of the Median (MoM) scores, which we used in all analyses [18].

Late in pregnancy (median 30.2 weeks of gestational age, 90% range 28.9 to 32.2) we performed Doppler velocimetry of the uterine arteries to measure the uterine artery resistance index (UtA-RI) as described previously [19]. Doppler measurements showed a high intraclass correlation coefficient value (> 0.80) with corresponding low coefficient of variation value (< 10%), which indicates adequate reproducibility [20].

Trained research assistants wearing usual clothing (i.e. no white coats) measured systolic and diastolic blood pressures in early, mid-, and late pregnancy in the right upper arm [21]. Before the measurement, women sat in an upright position with back support and relaxed for 5 min. The mean value of two blood pressure readings over a 60-s interval was documented.

### Pre-eclampsia

We obtained information on clinically diagnosed PE from cross-checked original hospital charts [22]. Given this study design (prospective cohort study), we defined the occurrence of PE according to the ISSHP criteria that were in effect at the time of the study, as new onset systolic blood pressure ≥ 140 mmHg and/or diastolic blood pressure ≥ 90 mmHg after 20 weeks of gestation and the presence of proteinuria with no evidence of urinary tract infection in a random urine sample [23].

### Six years after pregnancy

Six years (median 6.1 years, 90% range 5.6 to 7.6) after pregnancy blood pressure was measured with the validated automatic sphygmomanometer Datascope Accutorr Plus (Paramus, NJ, USA) [24]. Two-dimensional M-mode echocardiographic measurements were performed as described previously [25]. To assess aortic stiffness we used carotid-femoral pulse wave velocity (PWV) [26, 27]. Retinal vascular calibres were assessed by taking digital retinal photographs [28].

### Ten years after pregnancy

Ten years after pregnancy (median 9.8 years, 90% range 9.4 to 10.6) blood pressure was measured with the validated automatic sphygmomanometer Datascope Accutorr Plus (Paramus, NJ, USA) [24]. The common carotid artery (CCA) was measured using the ATL-Philips Model HDI 5000 (Seattle, WA, USA) or the Logiq E9 (GE Medical Systems, Wauwatosa, WI, USA) device 10 years after pregnancy. Carotid distensibility is a measure of carotid artery elasticity that has been introduced as a risk factor for cardiovascular disease [29]. Intima media thickness (IMT) is a measure of subclinical atherosclerosis associated with cardiovascular risk factors. Common carotid distensibility and IMT were assessed with the subjects in supine position, with the head tilted slightly to the contralateral side for the measurement in the common carotid artery. A region at 1.5 cm proximal to the origin of the bulb of the carotid artery was identified with the use of ultrasound. The end-diastolic diameter (D), the absolute stroke change in diameter during systole (ΔD), the relative stroke change in diameter (ΔD/D) and IMT were computed as the mean of three successive recordings from both left and right side. The cross-sectional arterial wall distensibility coefficient was calculated according to the following equation: distensibility coefficient = 2ΔD/(Dxpulse pressure) (10^− 3^ kPa). In a reproducibility study performed among 50 subjects, the intraclass correlation coefficient was 0.90 for distensibility and 0.91 for IMT.

### Covariates

We obtained information on maternal age, ethnicity, educational level, parity, smoking and method of conception through questionnaires repeatedly applied during pregnancy. At study enrolment during pregnancy, we measured maternal height (cm) and weight (kg) without shoes. Body mass index (BMI) was calculated (kg/m^2^). Community midwifery and hospital registries provided information on gestational age at birth, birth weight and placental weight.

### Statistical analysis

In this study, all available women with twin pregnancies (*n* = 91) were compared to all women with singleton pregnancies (*n* = 8107). Independent Student’s t, Mann-Whitney U and Chi-square tests were performed to test the differences in baseline characteristics between women with singleton and twin pregnancies.

### Pregnancy

Linear regression analyses were performed to relate women with singleton or twin pregnancies to placental biomarkers, blood pressure, uterine artery Doppler measurements, and cardiovascular outcomes at follow-up. Logistic regression analyses were performed to relate women with singleton or twin pregnancies to PE. For placental biomarkers, blood pressure in pregnancy and uterine artery Doppler measurements basic models were adjusted for maternal age and gestational age at time of measurement. If MoMs were used, measurements were not additionally adjusted for gestational age at time of measurement. In the multivariate analyses, we additionally adjusted for ethnicity, educational level, parity, smoking, method of conception and BMI at intake. For PE, the basic model was adjusted for gestational age at birth. To examine the role of the placenta, placental weight was added to the placenta model, and the mediating role of early pregnancy sFLT-1 and PGF as proxy for placental function were used. We analysed the direct causal mediation effects through mediation analyses. To explore blood pressure trajectories in pregnancy between women with singleton and twin pregnancies repeated measurement regression models were performed using the mixed model procedure with maternal blood pressure as repeated outcome measure. These models take the correlation between repeated measurements of the same woman into account.

### Maternal outcomes years after pregnancy

For outcomes 6 and 10 years after pregnancy, a linear regression basic model was adjusted for maternal age and interval time. The multivariate analyses were additionally adjusted for ethnicity, educational level, parity, smoking, method of conception and BMI at intake. When assessing retinal arteriolar calibre, we additionally adjusted for retinal venular calibre and vice versa. PWV was additionally adjusted for pulse at the time of PWV assessment.

Missing values in covariates were multiple-imputed, by using Markov chain Monte Carlo approach [30]. Five imputed data sets were created and analysed together. Statistical analyses were performed using Statistical Package of Social Sciences version 21.0 for Windows (SPSS, IBM Corp., Armonk, NY, USA), Statistical Analysis System version 9.4 (SAS, Institute Inc., Cary, NC, USA) and R version 3.5.0 (R Foundation, Vienna, Austria).

## Results

Table 1 shows maternal characteristics during pregnancy and 6 and 10 years after pregnancy. Woman with twin pregnancies were more often of Western ethnicity, multiparous, older, and had more pregnancies conceived by ART (Table 1).

**Table 1:** Baseline characteristics (*n* = 8198)

	Singleton (*n* = 8107)	Twin (*n* = 91)	***P***-value
**Intake**			
Maternal age (years)	29.5 (5.3)	32.1 (4.4)	< 0.001
Non-Western ethnicity (%)	3660 (45.1)	27 (29.7)	0.01
Lower education (%)	4978 (61.4)	46 (50.5)	0.05
Nulliparous (%)	4761 (58.7)	44 (48.4)	0.04
Assisted conception (%)	539 (6.6)	14 (15.4)	0.001
BMI at intake (kg/m2)	24.1 (19.5 to 34.0)	24.1 (19.1 to 36.0)	0.37
Smoking during pregnancy (%)	2382 (29.4)	28 (30.8)	0.68
**Early pregnancy**			
sFLT (pg./mL)	5.1 (2.2 to 12.0)	8.1 (3.6 to 15.8)	< 0.001
PGF (pg./mL)	43.4 (17.5 to 157.7)	46.6 (16.2 to 226.2)	0.62
SBP (mmHg)	115.7 (12.4)	116.8 (12.0)	0.45
DBP (mmHg)	68.4 (9.6)	68.9 (10.1)	0.68
**Mid-pregnancy**			
sFLT (pg./mL)	5.0 (1.9 to 14.3)	9.5 (2.7 to 24.6)	< 0.001
PGF (pg./mL)	202.4 (89.1 to 525.4)	405.0 (108.4 to 1183.8)	< 0.001
SBP (mmHg)	116.8 (12.1)	118.9 (12.1)	0.11
DBP (mmHg)	67.3 (9.4)	67.5 (9.8)	0.82
**Late pregnancy**			
SBP (mmHg)	118.3 (12.1)	119.5 (12.1)	0.42
DBP (mmHg)	69.2 (9.4)	71.0 (11.5)	0.10
**Birth**			
Gestational age at birth (weeks)	40.1 (36.7 to 42.1)	37.0 (30.8 to 39.5)	< 0.001
Premature birth < 37 weeks (%)	442 (5.5)	45 (49.5)	< 0.001
Premature birth < 34 weeks (%)	113 (1.4)	13 (14.3)	< 0.001
Birth weight (g)	3398.9 (561.7)	2470.2 (620.4)	< 0.001
Weight placenta (g)	633.4 (147.6)	1016.5 (235.7)	< 0.001
Pre-eclampsia (%)	168 (2.1)	11 (12.1)	< 0.001
**Six years after pregnancy**			
Interval time (years)	6.1 (5.6 to 7.6)	6.1 (5.7 to 7.4)	0.75
BMI (kg/m2)	24.8 (19.8 to 36.0)	24.4 (19.5 to 39.1)	0.61
Central retinal arteriolar calibre (SDS)	145.3 (16.9)	143.5 (17.3)	0.56
Central retinal venular calibre (SDS)	206.9 (22.5)	206.3 (24.5)	0.89
Pulse wave velocity (m/s)	7.6 (1.1)	7.4 (0.9)	0.45
Fractional shortening	37.0 (4.9)	36.4 (4.6)	0.37
Aortic root diameter (mm)	27.7 (2.9)	27.8 (2.8)	0.98
Left atrial diameter (mm)	33.9 (3.8)	34.2 (3.8)	0.48
Left ventricular mass (g)	127.0 (84.0 to 187.0)	133.0 (88.2 to 200.0)	0.05
SBP (mmHg)	119.4 (13.1)	119.3 (13.7)	0.97
DBP (mmHg)	71.0 (10.1)	70.1 (10.5)	0.51
**Ten years after pregnancy**			
Interval time (years)	9.8 (9.4 to 10.6)	9.8 (9.3 to 10.4)	0.27
BMI (kg/m2)	24.9 (20.0 to 36.2)	24.9 (20.3 to 39.9)	0.63
IMT (mm)	0.6 (0.1)	0.6 (0.1)	0.22
Distensibility (10^−3^/kPa)	31.3 (18.8 to 49.1)	31.9 (18.6 to 51.4)	0.37
SBP (mmHg)	114.6 (12.8)	111.2 (13.4)	0.13
DBP (mmHg)	68.6 (8.2)	67.3 (9.3)	0.40

Data are represented as n (%), mean (SD) or as the median with the 90% range. Differences in baseline characteristics were tested using Student’s t, Mann-Whitney U and Chi-Square tests. *Abbreviations*: *BMI* Body mass index, *sFLT* Soluble fms-like tyrosine kinase 1, *PGF* Placental growth factor, *SBP* Systolic blood pressure, *DBP* Diastolic blood pressure, *IMT* Intima media thickness

### Pregnancy

Women with twin pregnancies had higher levels of sFLT-1 and PGF in early and mid-pregnancy (Table 2).

**Table 2:** Association of outcome measures in women with a previous singleton or twin pregnancy

		**Singleton** (*n* = 8107)	**Twin** (*n* = 91)			
			**sFLT MoM** β (95% CI)	**PGF MoM** β (95% CI)		
**Early pregnancy**	Basic model	*Reference*	0.61 (0.45 to 0.78)*	0.55 (0.35 to 0.76)*		
	Confounder model	*Reference*	0.64 (0.48 to 0.79)*	0.56 (0.36 to 0.77)*		
**Mid-pregnancy**	Basic model	*Reference*	1.06 (0.85 to 1.26)*	1.02 (0.87 to 1.17)*		
	Confounder model	*Reference*	1.10 (0.90 to 1.30)*	1.05 (0.90 to 1.20)*		
		**Singleton** (*n* = 5133)	**Twin** (*n* = 61)			
**Six years**			**Arteriolar retinal** **calibre** (SDS)	**Venular retinal** **calibre** (SDS)	**PWV** (m/s)	
	Basic model	*Reference*	−1.50 (−4.09 to 1.08)	1.95 (−1.51 to 5.42)	−0.21 (−0.59 to 0.18)	
	Confounder model	*Reference*	−1.43 (−6.51 to 3.65)	1.81 (−4.94 to 8.57)	−0.20 (−0.59 to 0.19)	
			**Aortic root** **diameter** (mm)	**Left atrial** **diameter** (mm)	**Left ventricular** **mass **(g)	**Fractional** **Shortening**
	Basic model	*Reference*	−0.24 (−0.60 to 0.12)	0.26 (−0.24 to 0.75)	7.65 (−0.25 to 15.56)	−0.81 (−2.05 to 0.43)
	Confounder model	*Reference*	−0.39 (−1.08 to 0.31)	0.15 (−0.72 to 1.02)	5.82 (−1.49 to 13.12)	−0.85 (−2.09 to 0.40)
			**SBP** (mmHg)	**DBP **(mmHg)		
	Basic model	*Reference*	−1.92 (−5.20 to 1.37)	−2.20 (−4.77 to 0.37)		
	Confounder model	*Reference*	−1.85 (−5.04 to 1.34)	−1.97 (−4.45 to 0.50)		
		**Singleton** (*n* = 4655)	**Twin** (*n* = 53)			
**Ten years**			**IMT **(mm)	**Distensibility** (10^−3^/kPa)	**SBP **(mmHg)	**DBP **(mmHg)
	Basic model	*Reference*	0.00 (−0.02 to 0.02)	2.53 (−0.31 to 5.36)	−4.44 (−8.87 to −0.01)*	−2.50 (−5.37 to 0.37)
	Confounder model	*Reference*	0.00 (−0.02 to 0.02)	2.10 (−0.67 to 4.87)	−3.38 (−7.54 to 0.78)	−1.96 (−4.75 to 0.84)

Values are regression coefficients with the 95% confidence interval (CI) and are based on linear regression models. Women with the use of antihypertensive medication were excluded for blood pressure analyses. Basic model: adjusted for maternal age at intake and interval time. Arteriolar retinal calibre is additionally adjusted for venular calibre and vice versa. PWV is additionally adjusted for pulse at the time of PWV assessment. Confounder model: basic model additionally adjusted for ethnicity, educational level, parity, smoking, method of conception, and BMI at intake. *Abbreviations*: *sFLT* Soluble fms-like tyrosine kinase 1, *PGF* Placental growth factor, *PWV* Pulse wave velocity, *IMT* Intima media thickness, *SBP* Systolic blood pressure, *DBP* Diastolic blood pressure, *BMI* Body mass index. * *p*-value < 0.05

In total, 179 women (2.2%) developed PE; 168 women (2.1%) with a singleton and 11 women (12.1%) with twin pregnancies. Of these 179 women, 20 (11.2%) had early onset PE (< 34 weeks of gestation). Women with twin pregnancies had a higher risk to develop PE (OR 3.63; 95% CI 1.76 to 7.48) compared to women with singleton pregnancies. The risk to develop PE was even higher if we took placental weight into account (OR 6.76; 95% CI 2.81 to 16.24). This was mediated by placental function represented by sFLT-1 and PGF levels in early pregnancy. Despite this mediation through placental function, women with twin pregnancies still had a higher incidence of PE (Table 3). Women with twin pregnancies and PE showed no difference in sFLT-1 and PGF compared to women with twin pregnancies without PE (data not shown).

**Table 3:** Association of pre-eclampsia in women with a singleton or twin pregnancy

		PE OR (95% CI)		Mediation OR (95% CI)
	Singleton	Twin		sFLT / PGF ratio
Basic model	*Reference*	2.90 (2.03 to 4.16)	Direct effect	2.18 (1.38 to 3.43)
Confounder model	*Reference*	3.63 (1.76 to 7.48)	Direct effect	2.63 (1.05 to 6.57)
Placenta model	*Reference*	6.76 (2.81 to 16.24)	Direct effect	4.35 (1.43 to 13.22)

Data are represented as the odds ratio (OR) with the 95% confidence interval (CI) and are based on logistic regression models. The represented direct effect is the OR of women with twin pregnancies to develop PE if you take the mediating role of sFLT and PGF in early pregnancy into account. Basic model: adjusted for maternal age at intake and gestational age at birth. Confounder model: basic model additionally adjusted for ethnicity, educational level, parity, smoking, method of conception and BMI at intake. Placenta model: confounder model additionally adjusted for placental weight. *Abbreviations*: *PE* Pre-eclampsia, *sFLT* Soluble fms-like tyrosine kinase 1, *PGF* Placental growth factor

**Fig. 1:**
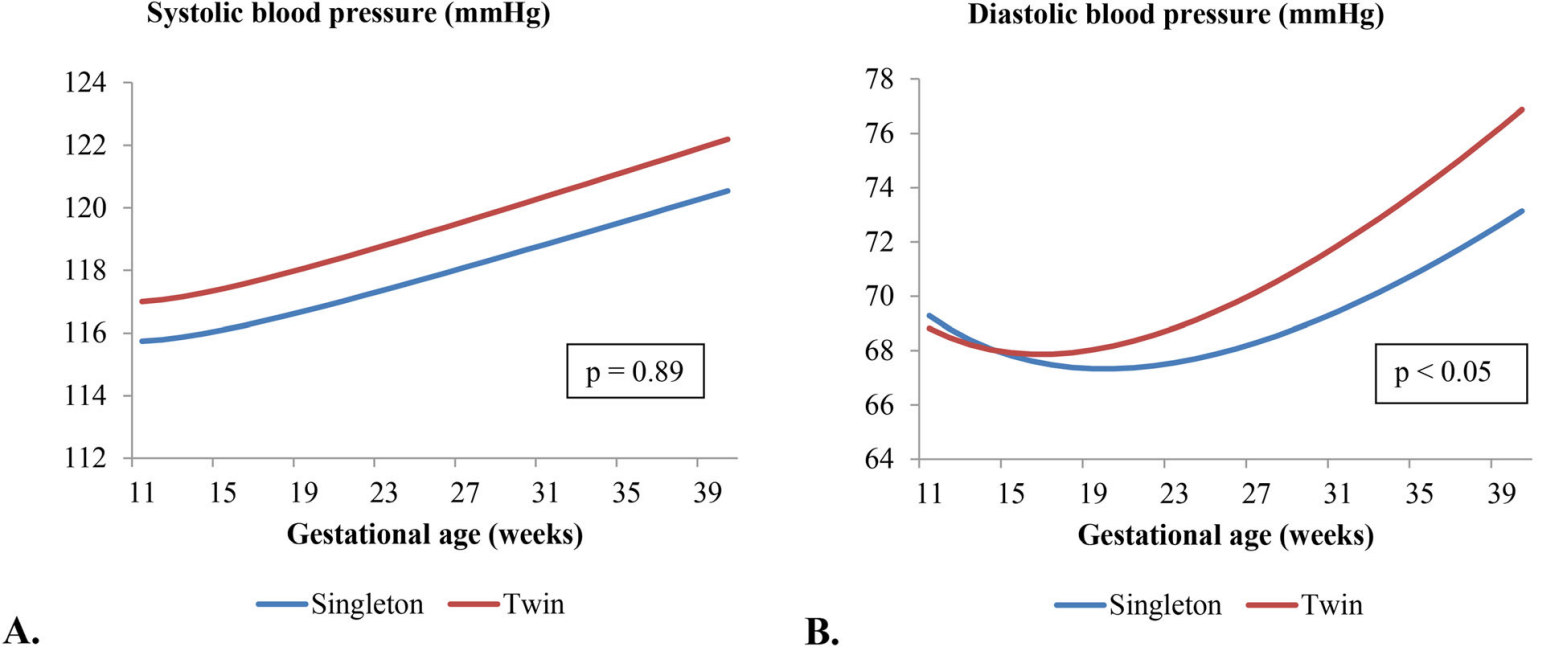
Blood pressure patterns in pregnancy stratified for women with singleton and twin pregnancies. **a** Change in systolic blood pressure in mmHg in women with singleton and twin pregnancies based on repeated measurements analyses. Data represent the unadjusted blood pressure pattern. Systolic blood pressure = β0 + β1 * pregnancy outcome + β2 * gestational age + β3 * gestational age ^ -2 + β4 * pregnancy outcome * gestational age. Women with the use of antihypertensive medication were excluded for blood pressure analyses (*n* = 11). **b** Change in diastolic blood pressure in mmHg in women with singleton and twin pregnancies based on repeated measurements analyses. Data represent the unadjusted blood pressure pattern. Diastolic blood pressure = β0 + β1 * pregnancy outcome + β2 * gestational age + β3 * gestational age ^ 0.5 + β4 * pregnancy outcome * gestational age. Women with the use of antihypertensive medication were excluded for blood pressure analyses (*n* = 11)

Figure 1 shows blood pressure patterns during pregnancy for women with twin pregnancies compared to women with a singleton pregnancies. A different DBP (*p* < 0.05), but not SBP (*p*-value 0.89) pattern was observed for women with twin pregnancies compared to women with singleton pregnancies with a cross-over in the DBP pattern around 15 weeks of gestation. This difference in DBP pattern was still observed after adjustment for confounders. Women with twin pregnancies and PE start with a higher DBP in early pregnancy compared to women with twin pregnancies without PE (*p* = 0.02). No difference in SBP or DBP pattern was observed.

### Maternal outcomes years after pregnancy

Twin pregnancies were not associated with SBP or DBP 6 years after pregnancy. Women with twin pregnancies had a lower SBP 10 years after pregnancy (β −4.44, 95% CI −8.87 to −0.01). After adjustment for confounders this association was no longer significant. Twin pregnancies were not associated with DBP 10 years after pregnancy (Table 2). Six years after pregnancy, no differences were found for women with twin pregnancies compared to women with singleton pregnancies regarding microvasculature, including arteriolar and venular retinal calibres. In addition, also no differences were found in macrovasculature 6 years after pregnancy, including PWV, left atrial diameter, aortic root diameter, fractional shortening and left ventricular mass. Ten years after pregnancy, no difference was found for IMT or distensibility as measure for elasticity in the CCA of women with twin pregnancies compared to women with singleton pregnancies (Table 2). We found no difference in SBP, DBP, retinal microvasculature, echocardiographic, and CCA measurements 6 and 10 years after pregnancy in women with twin pregnancies and PE compared to women with twin pregnancies without PE (data not shown).

## Discussion

Our study shows a different maternal adaptation to pregnancy in women with twin pregnancies compared to women with singleton pregnancies. Women with twin pregnancies have higher levels of sFLT-1 and PGF, a different DBP pattern in pregnancy and a higher risk for PE. Years after pregnancy, there seem to be no remaining cardiovascular differences in women with previous twin pregnancies compared to those with singleton pregnancies.

The increased risk of PE in women with twin pregnancies found in this study is in agreement with previous studies [4–6]. It was previously suggested that this increased risk was mediated through increased levels of sFLT-1 as a result of higher placental mass [9]. In our study the higher risk of PE in women with twin pregnancies could not be fully explained by higher placental mass, since women with twin pregnancies still show an increased risk of PE, even after taking placental weight, sFLT-1 and PGF into account. This suggests that the increased risk of PE in women with twin pregnancies is not merely the result of different placentation.

The average trajectory of blood pressure in pregnancy which decreases until mid-pregnancy and increases in late pregnancy, has been well described in women with singleton pregnancies [31]. For DBP, a different pattern was found in women with twin pregnancies compared to women with singleton pregnancies. We speculate a different physiologic basis for this difference. The higher DBP from mid-pregnancy onwards may be the effect of an increased intravascular volume, resulting in an increased DBP from mid-pregnancy onwards. Previously, Gaillard et al. showed that second to third trimester increase in BP is associated with an increased risk of gestational hypertensive disorders [32]. This is in agreement with our findings, showing that women with twin pregnancies have a higher increase in DBP from mid- to late pregnancy compared to women with singleton pregnancies resulting in a higher risk of PE.

Inadequate adaptation to pregnancy reveals diminished maternal reserves of women’s cardiovascular system. Therefore, pregnancy acts as a medical stress test for women [33]. It is thought that inadequate adaptation to pregnancy through endothelial dysfunction leads to a cascade of events that progresses to atherosclerosis and contributes to the risk of CVD [34]. Additionally, women at risk of CVD have risk factors as obesity, hyperlipidaemia and hypertension, also associated with endothelial dysfunction [35]. Since twin pregnancies demand a greater maternal adaptation, we hypothesized that women with twin pregnancies might have an increased risk of CVD by the increased risk of PE associated with persistent endothelial damage. Our study shows that women with twin pregnancies have a different adaptation during pregnancy compared to women with singleton pregnancies, but years after pregnancy these differences seem to resolve, not leading to an increased risk of CVD. Additionally we repeated the same analyses in women with twin pregnancies and PE compared to women with twin pregnancies without PE. No associations were found for women with twin pregnancies and PE. This may be explained by the relative small number of women with twin pregnancies in our study. In previous studies performed in women with singleton pregnancies, we did find associations years after pregnancy in women with PE compared to women without PE [28, 36]. However, it might also be that PE in women with twin pregnancies is potentially a different phenotype compared to PE in women with singleton pregnancies, independently from cardiovascular predisposition of mothers. Therefore we assume that PE and the link with CVD in women with singleton pregnancies can not be compared to PE in women with twin pregnancies. We hypothesize that the differences in adaptation during pregnancy in women with twin pregnancies are the result of the higher demand of both foetuses rather than failing pregnancy as cardiovascular stress test resulting in a higher risk of cardiovascular disease later in life.

### Strengths and limitations

A limitation is that the number of women with twin pregnancies in this study is rather low compared to the number of women with singleton pregnancies, therefore we performed the same analyses in a smaller number of selected women with singleton pregnancies (*n* = 199) matched on maternal age, ethnicity and level of education. Since this resulted in the same conclusions, we assume that the difference in group size did not influence our results. Due to incomplete information on chorionicity, no multivariate analysis was performed. Another limitation is that since this is a cohort study, measurements were restricted to standardized moments as part of the study design. We did not obtain outcomes of all women years after pregnancy. Six and ten years after pregnancy we obtained data from 63% at most. Nonresponse analyses showed that women with no attendance 10 years after pregnancy tended to be younger at intake, to be of non-Western descent, to have a lower level of education, to be more often nulliparous, to have a higher BMI and to smoke more often during pregnancy. They were also more likely to have an earlier gestational age at birth, more often delivered prematurely and their children had a lower birth weight (Table 4). If the selection mechanisms have been related to both determinant and outcome, this may have led to biased effect estimates. However, given the prospective nature of the study, this seems unlikely. It is impossible though, to exclude that this may influence our results. Another limitation is that we were restricted to pre-specified visiting moments. Therefore, 6 and 10 years after pregnancy may not be the best moments to measure cardiovascular adaptation. However, previous studies in women with singleton pregnancies did find differences for women with PE within this time period [28, 36].

**Table 4:** Maternal characteristics stratified for response ten years after pregnancy (n = 8198)

	Visit ten years after pregnancy (*n* = 4708)	No visit ten years after pregnancy (*n* = 3490)	***P***-value
**Maternal characteristics**			
Age at intake (years)	30.6 (4.9)	28.0 (5.4)	< 0.001
Non-Western ethnicity (%)	1734 (36.8)	1953 (56.0)	< 0.001
Lower education (%)	2483 (52.7)	2541 (72.8)	< 0.001
Nulliparous (%)	2900 (61.6)	1905 (54.6)	< 0.001
Assisted conception (%)	280 (5.9)	274 (7.9)	0.01
BMI at intake (kg/m2)	24.0 (19.6 to 33.2)	24.4 (19.3 to 35.0)	0.002
Smoking during pregnancy (%)	1198 (25.4)	1212 (34.7)	< 0.001
**Birth**			
Gestational age at birth (weeks)	40.1 (36.9 to 42.1)	40.0 (36.3 to 42.1)	< 0.001
Premature birth < 37 weeks (%)	241 (5.1)	246 (7.1)	< 0.001
Premature birth < 34 weeks (%)	58 (1.2)	68 (1.9)	0.01
Birth weight (g)	3411.0 (559.3)	3336.7 (601.0)	< 0.001
Weight placenta (g)	640.0 (153.7)	644.2 (168.4)	0.79

Data are represented as n (%), mean (SD) or as the median with the 90% range. Differences in baseline characteristics were tested using Student’s t, Mann-Whitney U and Chi-Square tests. *Abbreviations*: *BMI* Body mass index

## Conclusions

This study demonstrates that in pregnancy, differences in adaptation are present in women with a twin pregnancies compared to women with singleton pregnancies. These differences seem to resolve years after pregnancy. Therefore, women with twin pregnancies show no direct association with CVD later in life compared to women with singleton pregnancies.

## Supplementary information

**Additional file 1: Figure S1.** Flowchart showing the inclusion and exclusion criteria.

## Data Availability

Data requests can be made to the secretariat of Generation R.

## Abbreviations

ART: Assisted reproductive techniques
BMI: Body mass index
CCA: Common carotid artery
CI: Confidence interval
CVD: Cardiovascular disease
DBP: Diastolic blood pressure
IMT: Intima media thickness
OR: Odds ratio
MoM: Multiple of the Median
PE: Pre-eclampsia
PGF: Placental growth factor
PWV: Pulse wave velocity
SBP: Systolic blood pressure
SD: Standard Deviation
sFLT-1: Soluble fms-like tyrosine kinase 1
UtA-RI: Uterine artery resistance index
