# Supplementary figures and images for: Maternal cardiovascular adaptation to twin pregnancy: a population-based prospective cohort study
Source: BMC Pregnancy Childbirth. 2020 May 29;20:327. doi: 10.1186/s12884-020-02994-w (PMC7257120; doi:10.1186/s12884-020-02994-w)

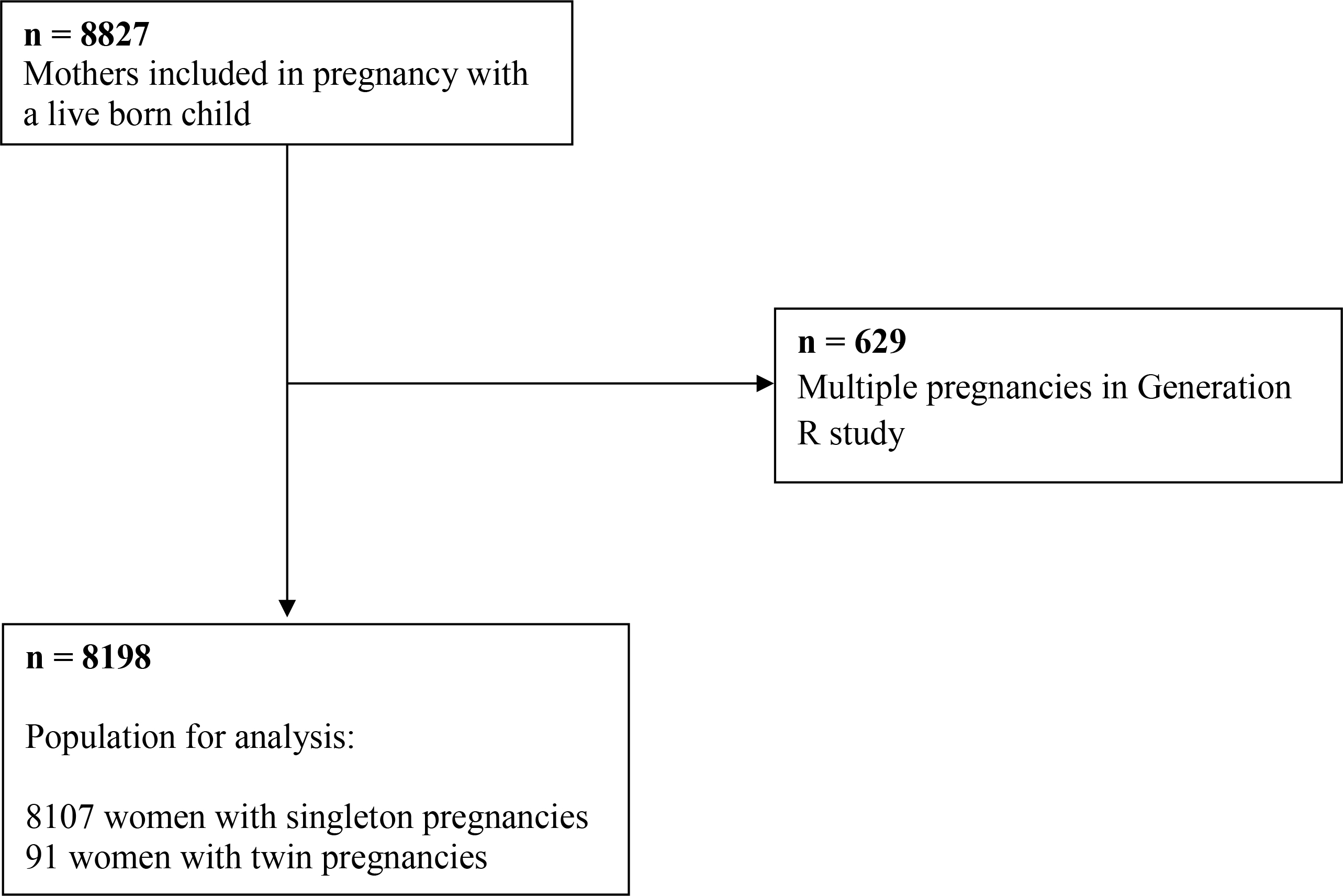

Supplement: Supplementary file 1 — Additional file 1: Figure S1. Flowchart showing the inclusion and exclusion criteria. [file 12884_2020_2994_MOESM1_ESM.jpg]
